# Deregulation of the MiR-193b-KRAS Axis Contributes to Impaired Cell Growth in Pancreatic Cancer

**DOI:** 10.1371/journal.pone.0125515

**Published:** 2015-04-23

**Authors:** Xianglan Jin, Yang Sun, Haiyan Yang, Ji Li, Shuangni Yu, Xiaoyan Chang, Zhaohui Lu, Jie Chen

**Affiliations:** Department of Pathology, Peking Union Medical College Hospital, Chinese Academy of Medical Sciences and Peking Union Medical College, Tsinghua University, Beijing, People’s Republic of China; Schulze Center for Novel Therapeutics, Mayo Clinic, UNITED STATES

## Abstract

Modulation of KRAS activity by upstream signals has revealed a promising new approach for pancreatic cancer therapy; however, it is not clear whether microRNA-associated KRAS axis is involved in the carcinogenesis of pancreatic cancer. Here, we identified miR-193b as a tumor-suppressive miRNA in pancreatic ductal adenocarcinoma (PDAC). Expression analyses revealed that miR-193b was downregulated in (10/11) PDAC specimens and cell lines. Moreover, we found that miR-193b functioned as a cell-cycle brake in PDAC cells by inducing G1-phase arrest and reducing the fraction of cells in S phase, thereby leading to dampened cell proliferation. miR-193b also modulated the malignant transformation phenotype of PDAC cells by suppressing anchorage-independent growth. Mechanistically, KRAS was verified as a direct effector of miR-193b, through which the AKT and ERK pathways were modulated and cell growth of PDAC cells was suppressed. Taken together, our findings indicate that miR-193b-mediated deregulation of the KRAS axis is involved in pancreatic carcinogenesis, and suggest that miR-193b could be a potentially effective target for PDAC therapy.

## Introduction

MicroRNAs (miRNAs) are a class of very small, non-coding RNAs that are evolutionarily conserved in many organisms. miRNAs suppress the expression of protein-coding genes in metazoans by binding to the 3′ untranslated region (3′-UTR) or even the coding region of their corresponding mRNA [1–3]. In this process, specific miRNAs pair-bond with target genes, leading to translational repression or/and mRNA destabilization [4]. Recent bioinformatic analyses have indicated that a great number of mRNAs are conserved target transcripts of miRNAs in mammals [5, 6]. miRNAs regulate a variety of cellular and developmental processes, including cell proliferation, survival, differentiation, animal development and disease [7–9]. Importantly, miRNAs display aberrant expression patterns in tumors and have emerged as important regulators of tumorigenesis and cancer progression, acting as tumor-suppressor genes or oncogenes [10, 11].

Pancreatic cancer is a malignancy with an extremely poor prognosis, with patients exhibiting dismal five-year relative survival rates of 6% [12]. This poor outcome is partly attributable to the inadequacy of currently available therapies and the fact that the cancer is usually diagnosed at late stages when these limited therapeutic options are no longer effective. Molecular alterations associated with pancreatic tumorigenesis and progression have been extensively investigated. Among the most frequent molecular alterations is KRAS, an oncogene that, when activated, causes cell growth and survival. Although KRAS mutations act as a key event in pancreatic carcinogenesis [13], targeting upstream signals that modulate KRAS activity may be a promising future approach for treating pancreatic cancer [14].

*In silico* screens using TargetScan (http://www.targetscan.org) have revealed that KRAS is targeted by the miRNA, miR-193b. In addition, Calin et al. found a high correlation between miRNA gene loci and cancer-associated genetic alterations [15]. Notable in this context, the miR-193b gene is located at 16p13, a region within chromosome 16 that exhibits genetic imbalance in pancreatic adenocarcinoma [16][17]. Moreover, aberrant expression of miR-193b has been detected in several human tumors [18–20] and miR-193b is associated with Mitogen-activated Protein Kinase (MAPK) signaling in pancreatic cancer [21]. However, the expression and function of miR-193b is not well characterized in pancreatic diseases.

In this study, we found that the expression of miR-193b was downregulated in pancreatic ductal adenocarcinoma (PDAC), the most common type of pancreatic cancer, compared to adjacent benign pancreatic tissue. In order to investigate the role of miR-193b in these disorders, we performed an *in vitro* gain-of-function analysis by transfecting cell lines with miR-193b mimics. miR-193b function in these cells was assessed by examining cell viability, proliferation, apoptosis and colony-formation ability, and the underlying molecular mechanism was probed by testing KRAS as a target of miR-193b.

## Materials and Methods

### Ethics statement

This study was approved by the Peking Union Medical College Hospital Institutional Review Board. Written informed consent was obtained from all the patients.

### Cell lines and pancreatic tissue samples

The pancreatic cancer cell lines, MIA PaCa-2, PANC-1, AsPC-1 and BxPC-3, and hTERT-HPNE (Human Pancreatic Nestin Expressing) human pancreatic duct epithelial cells were from American Type Culture Collection (ATCC). The type of hTERT-HPNE cells is “intermediary cells formed during acinar-to-ductal metaplasia” (according to ATCC information). All cell lines were cultured in complete growth medium containing Dulbecco's Modified Eagle's Medium (DMEM) and 10% fetal bovine serum (FBS) at 37°C in a humidified 5% CO_2_ atmosphere.

Cells were transfected with miRIDIAN hsa-miR-193b mimic or Negative Control #2 (Dharmacon, Thermo Fisher Scientific) at a final concentration of 25 nM using the transfection reagent DharmaFECT 4 (Dharmacon), according to the manufacturer’s instructions. Cell density was measured using a Countess Automated Cell Counter (Invitrogen).

Pancreatic tissue samples were collected from the Department of Pathology at Peking Union Medical College Hospital. Fresh samples were obtained from patients undergoing pancreatic resection and were immediately snap-frozen and stored in liquid nitrogen. In addition to histological confirmation of PDAC in paraffin-embedded sections immediately after surgery, the cancer status of PDAC and matched adjacent pancreas was reconfirmed in frozen sections of each specimen prior to RNA extraction. Patient characteristics, including sex, age, pathological diagnosis, differentiation and lymph node involvement, are presented in S1 Table.

### RNA quantitation and *in situ* hybridization

Total RNA was extracted from frozen tissue samples or cultured cell lines using TRIzol reagent (Life Technologies). RNA concentration was determined on a NanoDrop 1000 spectrophotometer (Thermo Fisher Scientific). miRNA was converted to cDNA using a TaqMan MicroRNA Reverse Transcription Kit (Applied Biosystems); reverse transcriptase-minus and no-template controls were included. Mature hsa-miR-193b expression levels were assessed by real-time polymerase chain reaction (PCR) using TaqMan MicroRNA assays (Applied Biosystems). Reactions were performed in triplicate on an IQ5 instrument (Bio-Rad) using the 2^-ΔΔCt^ method; U6 snRNA was used as an endogenous control.

A miRCURY LNA (locked nucleic acid) miRNA detection probe targeting hsa-miR-193b for *in situ* hybridization (No. 38611–15; Exiqon) was labeled with combined 5′ and 3′ double digoxigenin (DIG). The U6 positive control probe (No. 99002–01; Exiqon) and the scrambled negative control probe (No. 99004–01; Exiqon) were both 5′-DIG labeled. miRNA detection and localization using *in situ* hybridization were performed according to the manufacturer’s protocols [22]. Paraffin-embedded tissues from 22 cases of PDAC and one case of chronic pancreatitis were used.

### Western blotting and antibodies

Total protein was extracted from tissue samples using cold T-PER Tissue Protein Extraction Reagent or from cell lines using RIPA buffer containing Halt Protease Inhibitor Cocktail and Halt Phosphatase Inhibitor Cocktail (all from Thermo Scientific). Protein concentration in whole-cell extracts was determined with a Pierce BCA Protein Assay Kit (Thermo Scientific), following the manufacturer’s instructions. Proteins in extracts (60 μg protein) were separated by sodium dodecyl sulfate-polyacrylamide gel electrophoresis (SDS-PAGE) on 12% gels and transferred to a PVDF (polyvinylidene difluoride) membrane (Bio-Rad) for Western blot analysis. Primary antibodies against cyclin D1 (CCND1; 1:2000, #2926), cleaved PARP (1:1000, #9546), cleaved caspase-3 (1:500, #9661), ERK, p-ERK, Akt and p-Akt were purchased from Cell Signaling Technology. Primary antibodies against K-RAS (1:500, sc-30) and β-actin (1:5000, sc-47778) were from Santa Cruz Biotechnology.

### Cell vitality, proliferation, and cell cycle analysis

MIA PaCa-2 or PANC-1 cells were plated at 3000 cells/well in 96-well plates. One to four days after transfection, cell viability was assessed using a Cell Counting Kit-8 (CCK-8; Dojindo Molecular Technologies, Inc.). Cell proliferation was assessed with a BrdU (5-bromo-2′-deoxy-uridine) Labeling and Detection kit I (Roche), using miRNA mimics (GenePharma Co.). After cells were counterstained with 4',6-diamidine-2'-phenylindole dihydrochloride (DAPI; Roche), 6–8 representative pictures were taken from each specimen under an EVOS f1 fluorescence microscope (Advanced Microscopy Group). The percentage of BrdU-positive cells was calculated [23].

Pancreatic cells were harvested 24 hours after transfection and subjected to a cell-cycle analysis. Samples were fixed in 75% cold ethanol, digested with 200 μg/ml ribonuclease A (R6513, Sigma-Aldrich; stock solution: 10 mg/ml in 10 mM Tris-HCl pH 7.5, 15 mM NaCl), and stained with 50 μg/ml propidium iodide (PI; P4170, Sigma-Aldrich) [18, 24]. Cell cycle subcompartments were detected using a Beckman Coulter Epics XL flow cytometer.

### Apoptosis and colony-formation assays

Early-stage apoptosis was detected in MIA PaCa-2 and PANC-1 pancreatic cancer cells by first rinsing cells briefly with 0.125% trypsin (0.5×; GIBCO, Life Technologies) and then staining using a FITC Annexin V apoptosis Detection Kit I (BD Biosciences). Stained cells were analyzed on an Accuri C6 Flow Cytometer (BD Biosciences). Colony formation by cells treated with miRNA mimics (GenePharma Co.) was assayed as previously described [25].

### DNA constructs and luciferase reporter assay

A fragment of wild-type KRAS 3′-UTR was amplified by PCR from PANC-1 genomic DNA using TopTaq DNA Polymerase (QIAGEN) with the primers, 5′-GCT CTA GAA AGG CCA TTT CCT TTT CAC A-3′ and 5′-GCT CTA GAT GCA TGA CAA CAC TGG ATG A-3′ (underlined bases correspond to an *Xba*I restriction site). Antarctic phosphatase (New England Biolabs Inc.) was used to prevent self-cyclization. A luciferase reporter construct was prepared by digesting the PCR product and inserting it into the pGL3-Control Vector (Promega) at a site immediately downstream of the firefly luciferase gene. Reporter constructs containing mutated KRAS 3′-UTR, synthesized by Sangon Biotech, were also prepared. The miR-193b complementary sequence, prepared by annealing the two primers 5′-CTA GAA GCG GGA CTT TGA GGG CCA GTT T-3′ and 5′-CTA GAA ACT GGC CCT CAA AGT CCC GCT T-3′ (underlined bases correspond to an *Xba*I restriction site), was used to verify the transfection efficiency.

293A cells were co-transfected with luciferase constructs, pRL-TK, and miR-193b mimic or Negative Control #2 (50 nM; Dharmacon) using Lipofectamine 2000 (Invitrogen). Luciferase activity was determined 24 hours after transfection using the Dual-Luciferase Reporter Assay System (Promega, E1910) on a Modulus Microplate luminometer (Promega). The pRL-TK vector (Promega), expressing Renilla luciferase, was used to normalize for differences in transfection efficiency between samples.

### Statistical analysis

Experiments were repeated at least three times. The results were expressed as means ± SD. Differences were assessed with a two-tailed Student’s t test or Wilcoxon rank-sum test, and a p-value < 0.05 was considered statistically significant.

## Results

### miR-193b expression is downregulated in PDAC tissues and cell lines

**Fig 1:**
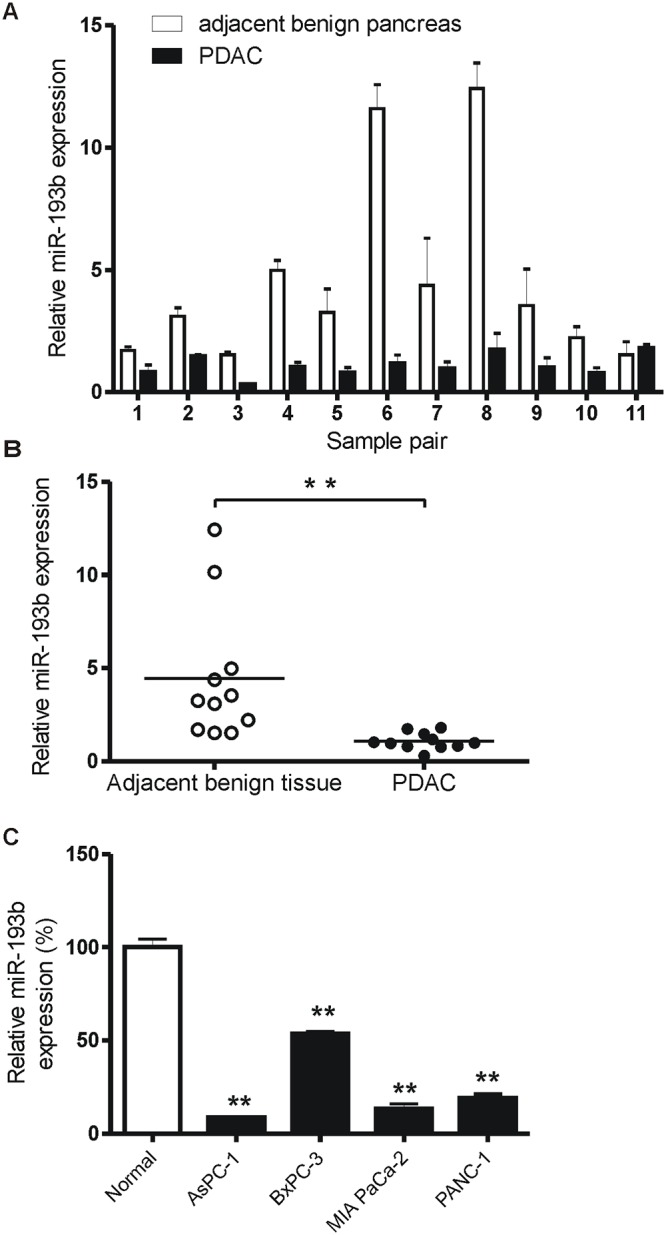
miR-193b is downregulated in PDAC tissues and cell lines. (A, B) Mature miR-193b expression levels were measured by real-time PCR in PDAC samples (n = 11) and matching adjacent benign pancreatic tissues (n = 11). Horizontal bars represent the mean value in each group (**p = 0.004, Wilcoxon rank sum test). (C) miR-193b expression pattern was quantified by real-time PCR in four pancreatic cancer cell lines and expressed relative to that in normal HPNE pancreatic cells (**p<0.01 vs. normal).

To explore the expression pattern of miR-193b in PDAC, we determined miR-193b levels in 11 surgically resected PDAC specimens with matched adjacent benign tissues. In addition to primary selection for PDAC based on paraffin-embedded sections, we reconfirmed the PDAC status by histological analysis of frozen sections of each specimen before RNA extraction (S1 Fig). Real-time PCR, used to analyze the expression levels of miR-193b, revealed that miR-193b expression was significantly decreased in 10 out of 11 PDAC samples compared to matched, adjacent benign tissues (Fig 1A and 1B). Overall, the expression levels of miR-193b in bulk PDAC samples were decreased 2.1- to 8.6-fold compared with adjacent normal tissues.

**Fig 2:**
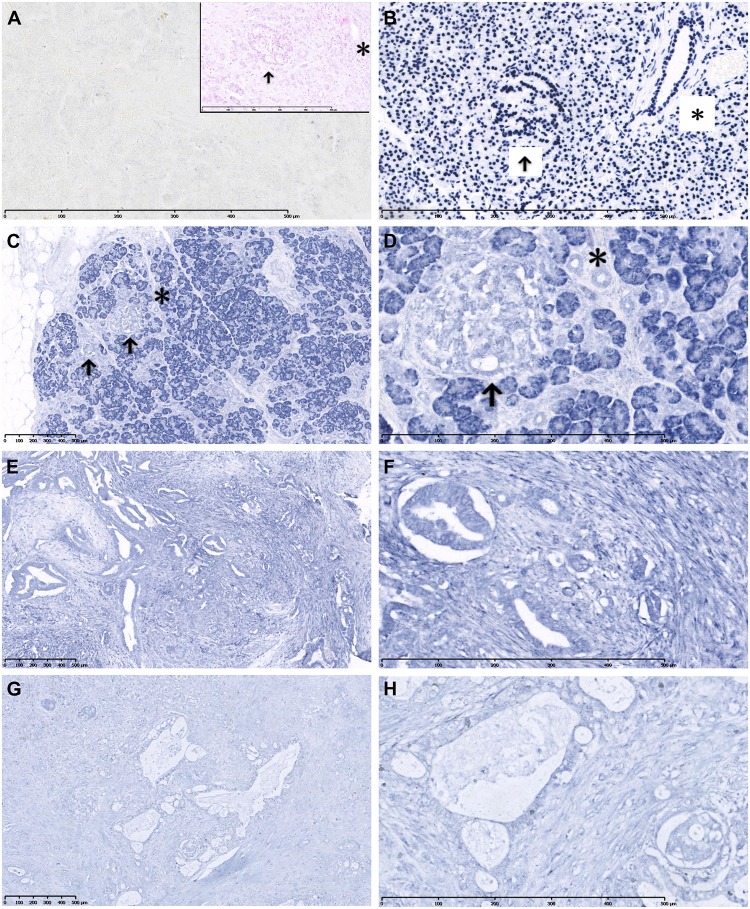
LNA *in situ* hybridization-based analysis of miR-193b in PDAC and matched adjacent benign tissues. (A) Negative control staining (scramble probe) of benign pancreas. To reveal structural details, this negative control was further stained with nuclear fast red (inset); islet (arrow) and ductal (*) cells are indicated. (B) Positive control (U6 probe) showing nuclear staining of all cells; islet (arrow) and ductal (*) cells are indicated. (C, D) Adjacent benign tissues showing strong positive miR-193b signals in acinar cells, but only weaker positive signals in islet (arrow) and ductal (*) cells. (E, F) A representative PDAC sample showing moderately positive (2+) labeling for miR-193b. (G, H) Another PDAC sample showing weakly positive (1+) labeling for miR-193b. D, F and H are four-fold zoom outs of C, E and G, respectively. Each horizontal bar represents 500 μm.

Since tumor tissues are a mix of many tumor cells and stroma cells, and benign tissues are predominantly made up of acinar cells. We further performed LNA *in situ* hybridization of paraffin-embedded PDAC specimens (n = 22). The expression of miR-193b was lower in PDAC samples than matched adjacent tissues (Fig 2; Table 1). In benign adjacent tissues, miR-193b was strongly (3+) positive in cytoplasm of acinar cells (20/22). Ductal cells that made up a small portion of benign tissues showed various staining levels from negative to moderately (2+) positive (Fig 2C and 2D). In PDAC tissues, no strong miR-193b expression was observed in tumor cells. 2+ miR-193b staining levels were detected in tumor cells, in 14 out of 22 cases (Fig 2E and 2F). 1+ miR-193b levels were seen in 8 out of 22 cases (Fig 2G and 2H).

**Table 1:** LNA *in situ* hybridization-based analysis of miR-193b expression in PDAC tissues (n = 22).

Category	Negative (-)	Weakly positive (+)	Moderately positive (++)	Strongly positive (+++)
PDAC	0	8	14	0
Adjacent benign acinar cells	0	0	2	20
Adjacent benign ductal cells	2	17	3	0

We next examined endogenous miR-193b expression levels in different pancreatic cancer cell lines. Compared with non-tumorigenic pancreatic duct hTERT-HPNE cells, miR-193b expression was significantly decreased in four different pancreatic cancer cell lines (Fig 1C).

Taken together, these data suggest that a decrease in miR-193b expression is a common feature of pancreatic cancer both *in vitro* and *in vivo*.

### miR-193b inhibits pancreatic cancer cell growth and proliferation

The reduced expression of miR-193b in pancreatic cancer led us to examine its functional role *in vitro*. Using a gain-of-function approach, we transfected MIA PaCa-2 and PANC-1 cells with miR-193b mimic or scrambled (negative control) oligonucleotide. Transfection efficiency was confirmed by quantitative PCR and luciferase reporter assays (S2 Fig).

**Fig 3:**
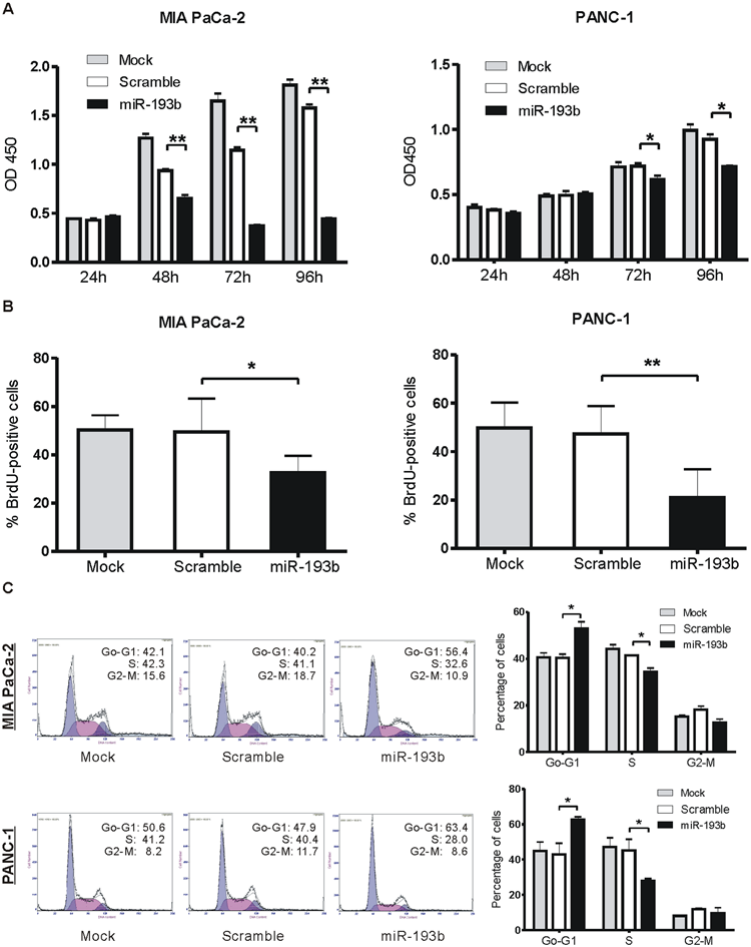
miR-193b overexpression inhibits cell proliferation and cell-cycle progression in PDAC cells. (A) MIA PaCa-2 and PANC-1 cells were transfected with 25 nmol/L miR-193b mimic or scrambled (negative control) oligonucleotide. Cell viability was determined on days 1 to 4 after transfection using CCK-8 assays. (B) Cell proliferation was assessed by BrdU incorporation assay 48 hours after transfection. (C) MIA PaCa-2 and PANC-1 cells transfected with miR-193b mimic or scrambled oligonucleotide were stained with PI and analyzed for cell-cycle distribution by flow cytometry (*p<0.05, **p<0.01).

The viability of cells was examined on days 1 to 4 using CCK-8 assays. Delivery of miR-193b mimics into cells significantly reduced the viability of both MIA PaCa-2 and PANC-1 cells compared with transfection of scrambled control miRNA (Fig 3A).

In order to study the mechanism underlying the miR-193b-induced decrease in cell viability, we further examined proliferation using BrdU-incorporation assays and evaluated cells for cell-cycle distribution. miR-193b overexpression suppressed the proliferative rate in both cell lines, reducing the percentage of BrdU-positive MIA PaCa-2 and PANC-1 cells by 16.8% and 26.4%, respectively, compared to scrambled controls (Fig 3B). Moreover, a cell-cycle analysis revealed that miR-193b overexpression induced a 12.5% increase in G1 phase and a 7.0% decrease in S phase cells in the MIA PaCa-2 cell line compared to scrambled controls; a similar increase in G1-phase cells (19.5%) and decrease in S-phase cells (17.3%) was observed in the PANC-1 cell line (Fig 3C). These results indicate that elevated expression of miR-193b in pancreatic cancer cells inhibits cell growth and cell-cycle progression by promoting G1-phase arrest and a subsequent reduction in the S-phase population.

### miR-193b overexpression is associated with apoptosis in MIA PaCa-2 and inhibits clonogenic potential

**Fig 4:**
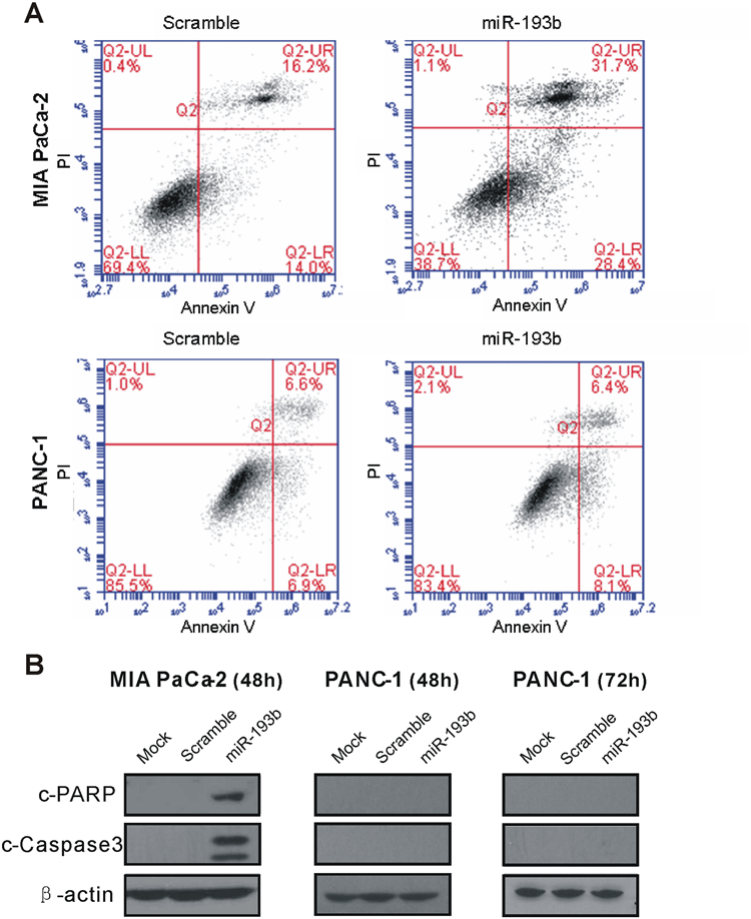
Effect of miR-193b on apoptosis in two pancreatic cancer cell lines. (A) MIA PaCa-2 and PANC-1 cells were transfected with miR-193b mimic or scrambled oligonucleotide; 24 hours later, cells were stained with PI and FITC Annexin V and analyzed by flow cytometry. Early apoptotic cells were identified as FITC Annexin V-positive and PI-negative. End-stage apoptotic and dead cells were FITC- and PI-positive. (B) MIA PaCa-2 and PANC-1 cells transfected with miR-193b mimic or scrambled oligonucleotide or mock-transfected (DhamaFECT 4 only) were subjected to immunoblot analysis using antibodies specific to c-PARP, c-caspase-3, or β-actin.

To determine whether miR-193b acts via an apoptotic mechanism, we treated MIA PaCa-2 cells with miR-193b mimic or scrambled oligonucleotide and tested for the presence of Annexin V-stained cells by flow cytometry. As expected, treatment of MIA PaCa-2 cells with the miR-193b mimic induced an increase in Annexin V-positive, early-apoptotic cells (Fig 4A). We also confirmed this result by determining the levels of the apoptosis-associated proteins, c-PARP and c-caspase-3 using Western blot analysis (Fig 4B). Interestingly, these assays showed no evidence of apoptosis induction in PANC-1 cells (Fig 4), suggesting a difference in the underlying signaling mechanisms in these two cell lines.

**Fig 5:**
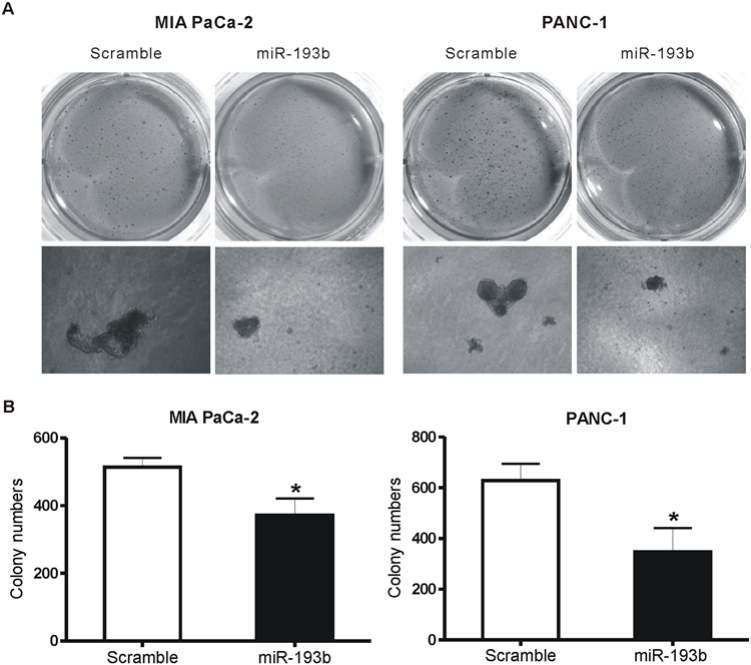
miR-193b inhibits anchorage-independent growth of PDAC cells in soft agar assay. Colony numbers were counted 2 weeks after plating of transfected MIA PaCa-2 and PANC-1 cells. (A) The upper panel was magnified by 1× and the lower panel was magnified by 40×. (B) The data is shown as mean±SD. (*p<0.05).

To investigate the effects of miR-193b on the anchorage-independent growth of pancreatic cancer cell lines, we transfected MIA PaCa-2 and PANC-1 with miR-193b mimic or scrambled oligonucleotide and performed colony-formation assays. After 2 weeks, there were fewer and smaller colonies in the miR-193b-overexpression group compared with the scrambled miRNA group in both cell lines (Fig 5).

### miR-193b downregulates KRAS expression by targeting its 3′-UTR

**Fig 6:**
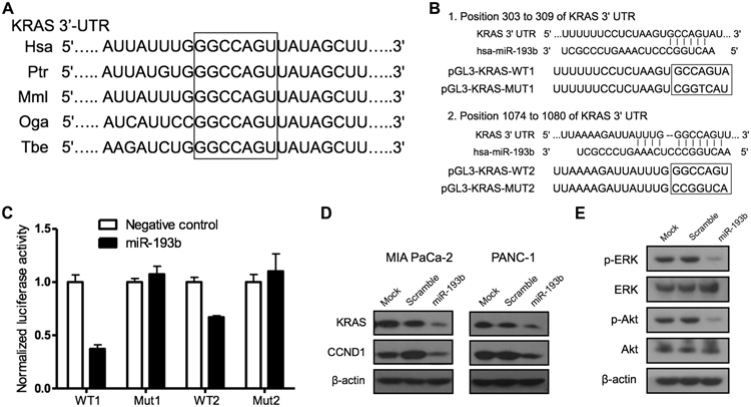
miR-193b regulates the expression of KRAS by directly targeting its 3′-UTR. (A) The TargetScan online database (http://www.targetscan.org) predicted KRAS as an miR-193b target. The site between 1074 and 1080 (boxed region), one of the two predicted miR-193b binding sites in the 3′-UTR of KRAS, is shown, highlighting the evolutionarily conservation among humans (hsa), the chimpanzees (Ptr), rhesus monkey (Mml), bushbaby (Oga), and treeshrew (Tbe). (B) The two miR-193b binding sites at positions 303 to 309 and 1074–1080 in the KRAS 3′-UTR, identified using the TargetScan online database (http://www.targetscan.org), are shown. Seed sequences were mutated as shown in the boxed regions. The wild-type or mutant constructs were inserted into the pGL3 vector directly downstream of the luciferase gene. (C) 293A cells were co-transfected with miR-193b or scrambled oligonucleotide, wild-type or mutant firefly luciferase constructs of the KRAS 3′-UTR segment containing miR-193b binding sites, and Renilla luciferase (endogenous control). Luciferase activity was measured 24 hours after transfection using the Dual-Luciferase Reporter Assay Systems. Renilla-normalized luciferase activity is expressed relative to that obtained for the scrambled oligonucleotide under each condition. WT, wild-type; Mut, mutant. (D) KRAS and CCND1 protein levels were assessed by Western blotting 48 hours after transfection. (E) The protein levels of p-ERK and p-Akt were evaluated by Western blotting 48 hours after miR-193b transfection.

To investigate the molecular mechanism of miR-193b, we first combined the computational approach, TargetScan (http://www.targetscan.org), with a consideration of oncogenes crucial in pancreatic carcinogenesis to identify putative target mRNAs. As shown in Fig 6A and 6B, computational analyses showed evolutionarily conserved miR-193b binding sites in the 3′-UTR of KRAS, which is known to be an important oncogene in pancreatic cancer.

We next assessed the functional interaction of miR-193b with KRAS using a luciferase reporter strategy. Constructs containing a segment of wild-type or mutant KRAS 3′-UTR inserted into the pGL-3 vector immediately downstream of the luciferase coding region were co-transfected with miR-193b mimic or scrambled miRNA into 293A cells; co-transfected pRL-TK plasmid served as an endogenous control. Whole-cell extracts were then obtained and assessed for luciferase activity using Dual-Luciferase Reporter Assays. These assays showed that transfection with miR-193b significantly decreased luciferase activity in cells co-transfected with wild-type KRAS 3′-UTR constructs compared with those co-transfected with mutant 3′-UTR controls (Fig 6C), verifying that KRAS is a direct target gene of miR-193b. Consistent with this, Western blot analyses showed that KRAS protein level was downregulated by miR-193b overexpression (Fig 6D); CCND1, a known target of miR-193b [18], was used as a positive control. Collectively, these data suggest that miR-193b suppresses KRAS expression by directly binding to its 3′-UTR.

Finally, we explored the downstream mechanisms underlying the antitumor effects of the miR-193b-KRAS axis. We found that restoration of miR-193b in PANC-1 cells triggered the downregulation of p-ERK and p-Akt (Fig 6E).

## Discussion

Pancreatic cancer—often diagnosed at late stages when a patient is beyond cure—is characterized by its lethal nature. It is therefore important to understand its cellular origin, which is still a matter of debate. It has been reported that acinar-ductal metaplasia (ADM) is associated with pancreatic intraepithelial neoplasia (PanIN) formation [26] and pancreatic carcinoma [27]. Recent reports have found that PDAC might originate from acinar cells and that KRAS induced acinar-to-ductal reprogramming plays a key role in PDAC initiation [28]. It has also been extensively reported that pancreatic cancer exhibits aberrant patterns of miRNA expression. In the present study, deregulated miR-193b was observed in PDAC samples, which prompted us to further investigate the role of miR-193b in the development of this disease.

Here, we found that miR-193b was downregulated in most (10/11) of the tested bulk specimens from PDAC patients compared to matched adjacent tissues. As tumor tissues comprise a mix of many different cell types, we further used LNA *in situ* hybridization (n = 22 samples) to visualize the expression pattern of miR-193b in detail. In general, the expression level of miR-193b was high in “normal” acinar cells (3+), but comparably lower in PDAC cells (1+-2+). To examine the potential involvement of miR-193b in the early stage of PDAC development, we assessed the expression of miR-193b in chronic pancreatitis (CP). In peritumoral CP-like changes, acinar cells were strongly positive for miR-193b, whereas other components (e.g., duct-like structures, stroma, and islets of Langerhans) showed relatively weaker staining; 20 of 22 samples exhibited downregulated miR-193b in duct-like tissues (S3A–S3D Fig). In a single case of CP without PDAC, the expression pattern of miR-193b was similar with CP adjacent to PDAC (S3E and S3F Fig).

CP is a noted risk factor for PDAC [29], and ADM or tissues in which acinar cells have dedifferentiated to ductal-like structures are prone to transformation [30]. Thus, our results implicate decreased miR-193b is involved in the early stage of PDAC carcinogenesis. This raises the question: What are the functional roles of miR-193b in chronic inflammation versus PDAC? There are two possible explanations. First, previous reports showed that elevated levels of KRAS signaling in acinar cells is a direct linker between CP and PDAC: high KRAS activity induces inflammation and fibrosis, which then progress to PDAC [31, 32]. In CP, KRAS may be upregulated when its inhibitor (miR-193b) is downregulated, and the higher KRAS activity could induce a series of inflammatory programs that lead to cancer. Second, accumulating evidence suggests that PDAC originates from acinar cells, and that the precursor lesion of PDAC (PanIN) occurs through a duct-like state in acinar cells [28, 30, 33]. Chronic irritation or KRAS mutation can cause ADM in the pancreas [34]. Furthermore, previous studies indicated that KRAS and MAPK are required for the development of ADM and PanIN in mice [35, 36], and MAPK was found to be upregulated in pancreatitis [37]. Here, we show that miR-193b is downregulated in duct-like structures and PDAC. KRAS is directly targeted by miR-193b, leading to regulation of the KRAS effector, MAPK. Thus, miR-193b might be involved in sustaining inflammation-induced ADM, which is prone to tumorigenesis. Further studies are needed to clarify the underlying mechanism. However, we speculate that miR-193b is downregulated during the formation of PDAC from acinar cells via ADM, and that this alteration, along with changes in other factors, contributes to acinar cell differentiation and finally PDAC carcinogenesis.

Many previous studies found that KRAS oncogene is mutated in more than 95% of PDAC tissues. Our present study observed that miR-193b was frequently decreased in PDAC samples. In PDAC cell lines, we found that miR-193b expression is higher in BxPC-3 cells bearing wild-type KRAS compared to the AsPC-1, MIA PaCa-2 and PANC-1 cells, which harbor mutant KRAS (mutation statuses were obtained from www.atcc.org and www.sanger.ac.uk). Based on these findings, we speculate that the KRAS mutation status may be similar in patient materials and pancreatic cancer cell lines. This would further suggest that the downregulation of miR-193b may be associated with the mutation status of KRAS. But how do oncogenic KRAS mutations affect the function of miR-193b in PDAC development?

A group searching for MAPK-regulated microRNAs in pancreatic cancer cells found that MAPK activation negatively regulated miR-193b [21]. It is also reported that MAPK activity is sustained in KRAS mutant mice, but transient in wild-type mice [35]. Our data showed that miR-193b directly targets KRAS and thereby modulates ERK signaling. Taken together, these findings suggest that a KRAS-MAPK-miR-193b positive feedback loop may contribute to upregulating KRAS during pancreatic tumorigenesis in the presence of mutant KRAS. Furthermore, given that KRAS mutation is the initiating event in PDAC, mutant KRAS probably attenuates miR-193b expression, thereby contributing to KRAS-driven PDAC tumorigenesis. Similarly, KRAS mutation inhibits the expression of miR-143/145 through RREB1, thereby increases the level of KRAS (miR-143/145 target), forming a positive feedback loop affecting KRAS pathway [38]. The presence of inflammatory irritants plus KRAS mutation can induce the positive feedback circuit mediated by NF-κB signaling, triggering a sustained and pathological up-regulation of KRAS and result in inflammation and PanIN [39]. These kinds of positive feedback mechanisms might be universal in sustaining high levels of oncogenic KRAS activity, which then drive the initiation and development of PDAC [40]. Further investigations are needed to address the impact of mutant versus wild-type KRAS on miR-193b activity in PDAC cells, and to clarify the mechanisms underlying the interaction of KRAS with miR-193b.

Restoration of miR-193b in pancreatic cancer cells induced a remarkable array of biological processes, including inhibition of proliferation, cell-cycle arrest, and suppression of colony formation. We also found that the beginnings of reduced miR-193b levels compared with normal acinar cells were detected in duct-like changes around tumor. These data suggest that miR-193b downregulation and the miR-193b-KRAS axis might be involved in PDAC initiation. Using luciferase reporter assays, we verified that KRAS is indeed a direct target of miR-193b. Western blot analyses further showed that overexpression of miR-193b decreased KRAS levels, consistent with an effector role of KRAS in PDAC cells/tissues with downregulated miR-193b expression.

In 2007, two groups used microarray analysis to reveal differentially expressed miRNAs in pancreatic adenocarcinoma, chronic pancreatitis, and normal pancreas [41, 42]. They found that several miRNAs were decreased in pancreatic cancers compared to chronic pancreatitis and normal pancreas. Greither et al. identified four miRNAs that correlated with poorer survival of pancreatic cancer patients [43]. It has been shown that miR-193b is downregulated in other cancers in addition to pancreatic cancer, such as melanoma [18], non-small-cell lung carcinoma [44], hepatic cell carcinoma [19], and endometrial adenocarcinoma [20]. Thus, miR-193b appears to exhibit reduced expression levels in diverse cancers compared to matching benign tissues, suggesting a universal effect of this miRNA in cancer development. In addition, miR-193a and miR-193b shared the same seed sequence and targets such as PLAU and KRAS [45]. However, they differ in chromosomal locations and non-seed nucleotide sequences; potential non-redundancy among miRNA families is still unclear.

Interestingly, miR-193b was found to significantly induce apoptosis in MIA PaCa-2 cells but not in PANC-1 cells. This result, which was confirmed by different methods (Western blot detection of the apoptosis markers c-PARP and c-caspase-3, flow cytometry analysis of the Annexin V staining), suggests different underlying apoptotic mechanisms in these two cancer cell lines. Our interpretation of these findings is that miR-193b dampens pancreatic cell proliferation, mainly by acting as a brake on the cell cycle.

Downstream effectors of KRAS signaling in pancreatic cancer were widely explored. Among them, Ras/Raf/ERK and Ras/PI3K/AKT signals are major effector pathways in PDAC tumorigenesis [46], especially through cell cycle regulation [47]. Our colleagues have previously demonstrated that other miRNAs (miR-96, miR-217 and miR-27a) function in pancreatic cancer by directly or indirectly affecting KRAS activity, a process that involves PI3K/AKT or RAF-MEK-ERK signaling cascades [25, 48, 49]. To explore the downstream mechanisms underlying the antitumor effects of the miR-193b-KRAS axis, we found that both p-ERK and p-AKT were downregulated in miR-193b-treated PDAC cells. Recent findings showed that Ras/Raf/ERK could function with AKT synergistically in genesis and maintenance of PDAC [50]. Thus, key effectors in KRAS signaling may be tightly regulated by miR-193b in pancreas, and miR-193b restoration could target Ras/Raf/ERK and Ras/PI3K/AKT to suppress PDAC growth.

In our work, Cyclin D1 showed decreased level after miR-193b treatment in PDAC cells. Ras-dependent Cyclin D1 expression relies on sustained RAF/MEK/ERK activation, and correspondingly affects G1 to S phase transition [51]. miR-193b directly targeted to KRAS and subsequently caused ERK pathway deregulation, may contribute to Cyclin D1 downmodulation, resulting in dampened cell cycle progression. Interestingly, previous reports showed that miR-193b repressed melanoma cell proliferation by directly targeting Cyclin D1 [18]. Actually, a group of targets concurrently modulated by a single miRNA make up the biological network, resulting in miRNA-associated functions [52]. Therefore, other unknown functional molecules targeted by miR-193b, maybe together contribute to the tumor-inhibiting effect, which might arouse interesting further exploration in revealing the comprehensive network of miR-193b-KRAS axis in PDAC.

Collectively, our findings demonstrate that miR-193b is frequently downregulated in PDAC samples and has potential tumor-suppressor activity. Dysregulation of the miR-193b-KRAS axis appears to be involved in pancreatic carcinogenesis, indicating that miR-193b is a potentially new therapeutic target in PDAC.

## Supporting Information

S1 Fig Hematoxylin and eosin staining of frozen sections of PDAC specimens. The status of each section was histologically confirmed by senior pathologists before RNA and protein extraction.
(TIF)

S2 Fig Verification of miR-193b transfection efficiency by real-time PCR and luciferase assays. (A) Relative expression of miR-193b in MIA PaCa-2 and PANC-1 cells transfected with 25 nmol/L miR-193b mimic or scrambled oligonucleotide for 48 hours. (B) 293A cells were co-transfected with 50 nmol/L miR-193b mimic and a pGL3 vector containing a complementary miR-193b (c-miR-193b) segment; co-transfected pRL-TK served as a control for transfection efficiency. Upregulation of miR-193b suppressed the luciferase activity of pGL3-c-miR-193b.
(TIF)

S3 Fig LNA *in situ* hybridization-based analysis of miR-193b expression in CP-like regions adjacent to PDAC (two representative cases) and CP without PDAC (one case). Duct-like tissues are particularly prominent. (A, B) A case with peritumoral CP-like changes: miR-193b staining is lower in duct-like structures compared to acinar cells. (C, D) A second case with peritumoral CP-like changes: miR-193b staining is lower in the tubular complex compared to the acinar cells. (E, F) A case of CP without PDAC: miR-193b staining is lower in the duct-like tissues compared to the acinar cells. B, D and F are magnifications of A, C and E, respectively. Horizontal bar represents 500 μm.
(TIF)

S1 Table Characteristics of patients who underwent resection for PDAC. (XLSX)
