# Supplementary figures and images for: Deregulation of the MiR-193b-KRAS Axis Contributes to Impaired Cell Growth in Pancreatic Cancer
Source: PLoS One. 2015 Apr 23;10(4):e0125515. doi: 10.1371/journal.pone.0125515 (PMC4408116; doi:10.1371/journal.pone.0125515)

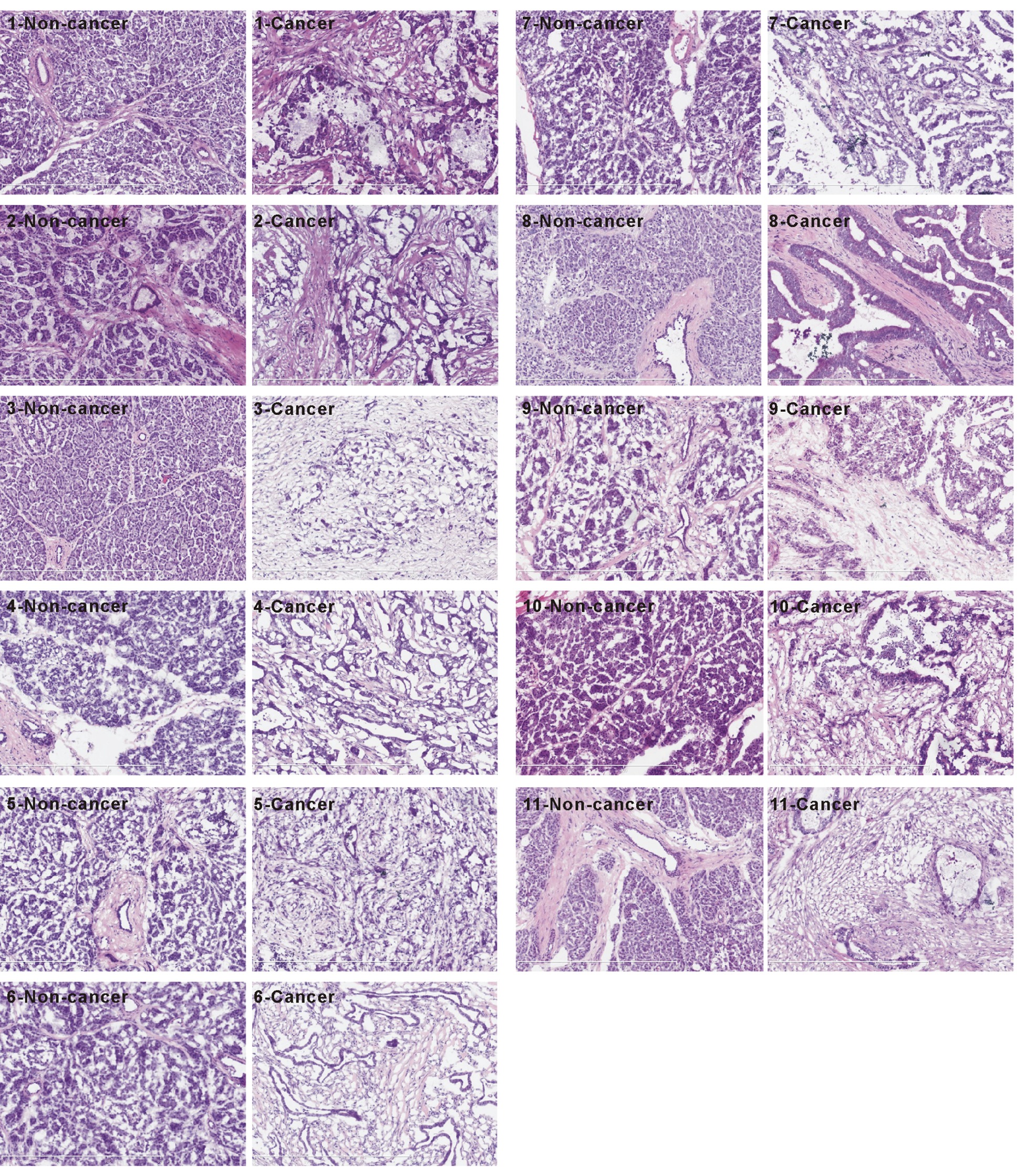

Supplement: S1 Fig — The status of each section was histologically confirmed by senior pathologists before RNA and protein extraction. (TIF) [file pone.0125515.s001.tif]

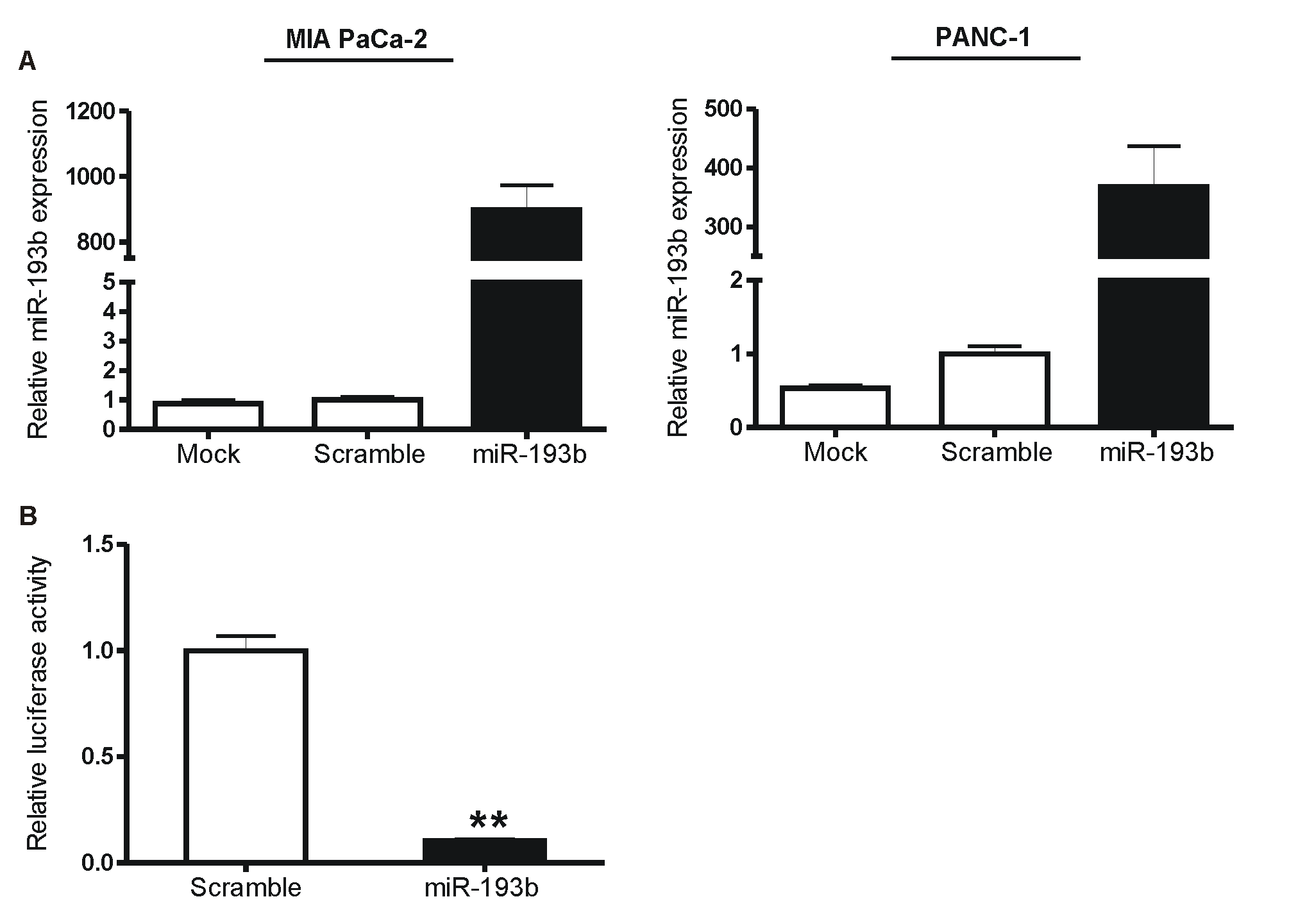

Supplement: S2 Fig — (A) Relative expression of miR-193b in MIA PaCa-2 and PANC-1 cells transfected with 25 nmol/L miR-193b mimic or scrambled oligonucleotide for 48 hours. (B) 293A cells were co-transfected with 50 nmol/L miR-193b mimic and a pGL3 vector containing a complementary miR-193b (c-miR-193b) segment; co-transfected pRL-TK served as a control for transfection efficiency. Upregulation of miR-193b suppressed the luciferase activity of pGL3-c-miR-193b. (TIF) [file pone.0125515.s002.tif]

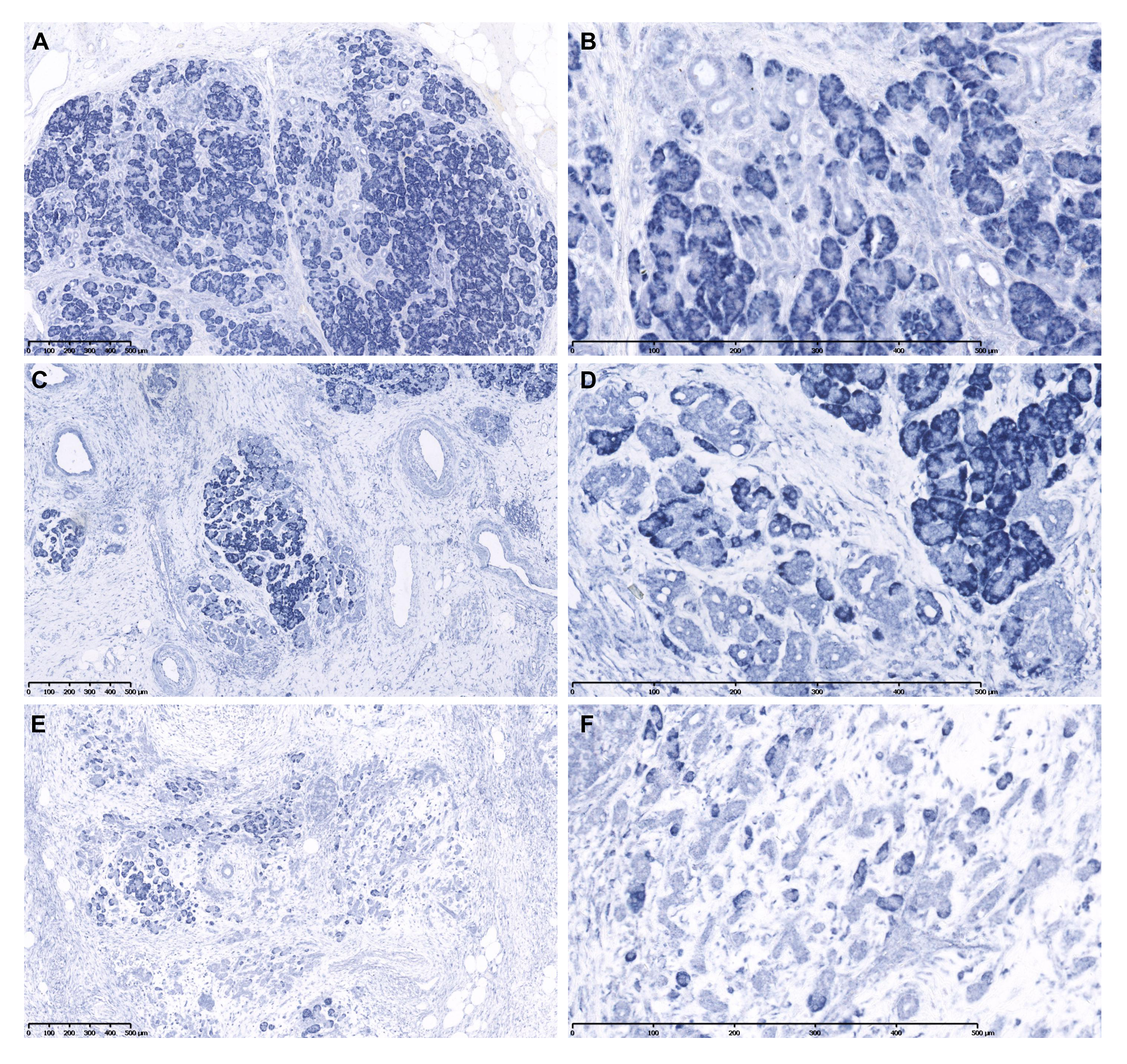

Supplement: S3 Fig — (A, B) A case with peritumoral CP-like changes: miR-193b staining is lower in duct-like structures compared to acinar cells. (C, D) A second case with peritumoral CP-like changes: miR-193b staining is lower in the tubular complex compared to the acinar cells. (E, F) A case of CP without PDAC: miR-193b staining is lower in the duct-like tissues compared to the acinar cells. B, D and F are magnifications of A, C and E, respectively. Horizontal bar represents 500 μm. (TIF) [file pone.0125515.s003.tif]
